# Nomogram based on computed tomography fractal dimension for predicting spread through air spaces in lung adenocarcinoma

**DOI:** 10.3389/fmed.2026.1772475

**Published:** 2026-03-11

**Authors:** Jiayu Ma, Xiaomeng Shi, Wei Ren, Yan Li, Fang Wang, Lili Yang

**Affiliations:** 1 Medical Imaging Center, People’s Hospital of Ningxia Hui Autonomous Region, Ningxia Medical University, Yinchuan, Ningxia Hui Autonomous Region, China; 2 CT Imaging Research Center, GE Healthcare China, Beijing, China

**Keywords:** computed tomography, fractal dimension, lung adenocarcinoma, nomogram, predictive model, tumor spread through air spaces

## Abstract

**Purpose:**

This study aimed to develop and validate a CT-based nomogram incorporating three-dimensional fractal dimension (FD 3D) to noninvasively predict tumor spread through air spaces (STAS) in stage IA lung adenocarcinoma.

**Materials and methods:**

A retrospective analysis was performed on 110 patients with stage IA lung adenocarcinoma who underwent surgical resection. CT morphological features and fractal-dimension metrics were collected. Patients were categorized into STAS-positive (*n* = 48) and STAS-negative (*n* = 62) groups based on pathology. Univariate and multivariate logistic regression analyses were conducted to identify independent predictors of STAS. Receiver operating characteristic (ROC) curve analysis evaluated predictive performance, and a nomogram model was constructed and internally validated. Based on the nomogram score, patients were further stratified into low- and high-risk STAS groups using the optimal cutoff value determined by the maximum Youden index.

**Results:**

Univariate analysis showed significant differences in consolidation-to-tumor ratio (CTR) (*p* < 0.001), morphological irregularity (*p* = 0.006), lobulation (*p* = 0.039), pleural indentation (*p* = 0.004), vascular convergence (*p* = 0.010), and FD 3D (*p* < 0.001) between groups. Multivariate analysis identified CTR, morphological irregularity, lobulation, and FD 3D as independent predictors of STAS in stage IA lung adenocarcinoma. The nomogram model achieved an area under the curve (AUC) of 0.894 (95%CI: 0.821–0.944; *p* < 0.001), with a sensitivity of 75.00% and a specificity of 90.32%. At the optimal cutoff value of 0.56, the model demonstrated a positive predictive value (PPV) of 85.71% in the high-risk group (*n* = 42, 38.18%) and a negative predictive value (NPV) of 82.35% in the low-risk group (*n* = 68, 61.82%), with significant differences in STAS prevalence between groups (85.71% vs. 17.65%, *χ*^2^ = 46.18, *p* < 0.001).

**Conclusion:**

The CT-based nomogram integrating FD 3D and key imaging features can noninvasively predict STAS status in stage IA lung adenocarcinoma. This model shows promise for assisting surgical decision-making, though prospective studies are needed to validate its clinical utility.

## Highlights

- Three-dimensional fractal dimension (FD 3D) was introduced for STAS prediction.
- FD 3D quantifies tumor spatial complexity and correlates with invasive behavior.
- A CT-based nomogram integrating FD 3D achieved an AUC of 0.89, with 85.71% PPV and 82.35% NPV in risk-stratified analysis.
- The model demonstrated good calibration and favorable clinical utility at the optimal cutoff of 0.56.
- The nomogram shows promise for assisting surgical decision-making, pending prospective validation.

## Introduction

According to data from the Global Cancer Observatory, the incidence and mortality rates of lung cancer are steadily increasing, making it the leading cause of cancer-related deaths worldwide, with adenocarcinoma being one of the most prevalent types of lung cancer (1, 2). In 2015, Kadota et al. (3) first introduced the concept of tumor spread through air spaces (STAS) in their study of pathological features. They described the diffusion of tumor cells in alveolar spaces, noting that these cells can appear as micropapillary clusters, solid nests, or single cells beyond the tumor edge. Subsequently, WHO classified it as a new type of invasive lung adenocarcinoma, akin to vascular and lymphatic invasion, and deemed it to have a significant impact on the prognosis of lung cancer (4). A study examining the prognostic significance of STAS across various surgical types and recurrence sites in 735 Japanese patients who underwent lung adenocarcinoma resection found that, among stage I STAS patients, sublobar resection was linked to a higher risk of both systemic and local recurrence compared to lobectomy (5). Notably, this finding is especially relevant for stage IA lung adenocarcinoma, a subgroup in which preoperative STAS assessment is particularly critical for surgical decision-making.

Currently, no parameters have been established in the clinical field to accurately predict the presence of STAS before surgery. Multiple studies have demonstrated that preoperative frozen sections are not effective in indicating a positive status for STAS, exhibiting low sensitivity and positive predictive value, which limits their widespread clinical application (6, 7). Fractal dimension (FD) is a quantitative measure that describes the complexity of a fractal, primarily reflecting how effectively a complex shape occupies space and serving as an indicator of the irregularity of intricate forms. It can be applied to analyze both two-dimensional (2D) and three-dimensional (3D) images. A retrospective study evaluated tumor heterogeneity in computed tomography (CT) images using FD and filtered-histogram texture analysis. Deng et al. (8) conducted a retrospective analysis of 135 patients with stage N0 non-small cell lung cancer and found that two-dimensional fractal dimension (FD 2D) was an independent predictor of lymphovascular invasion (odds ratio [OR]:0.01, *p* = 0.033), significantly improving the accuracy and reliability of the predictive model. The aforementioned studies have demonstrated the significant value of FD 2D in predicting clinical issues; however, the specific utility of three-dimensional fractal dimension (FD 3D) lesions in predicting STAS in lung adenocarcinoma has not been thoroughly investigated.

While previous studies have mainly focused on radiomics signatures or conventional CT morphological features to predict STAS, these approaches primarily capture intensity or texture variations on 2D slices. In contrast, FD analysis quantifies the intrinsic spatial complexity and self-similar structure of the tumor, reflecting its three-dimensional growth heterogeneity.

Unlike radiomics-based STAS prediction models that rely on high-dimensional texture features, the present study focuses on three-dimensional fractal dimension as a geometry-oriented descriptor of tumor spatial complexity, aiming to capture growth discontinuity that is pathologically characteristic of STAS. Therefore, the present study introduces FD 3D into the prediction of STAS for the first time, aiming to bridge the gap between radiomic texture and spatial invasiveness. By integrating FD 3D with key CT morphological features, we propose a novel non-invasive nomogram to identify high-risk STAS before surgery and guide individualized surgical strategies.

## Materials and methods

### Patient grouping

**Figure 1:**
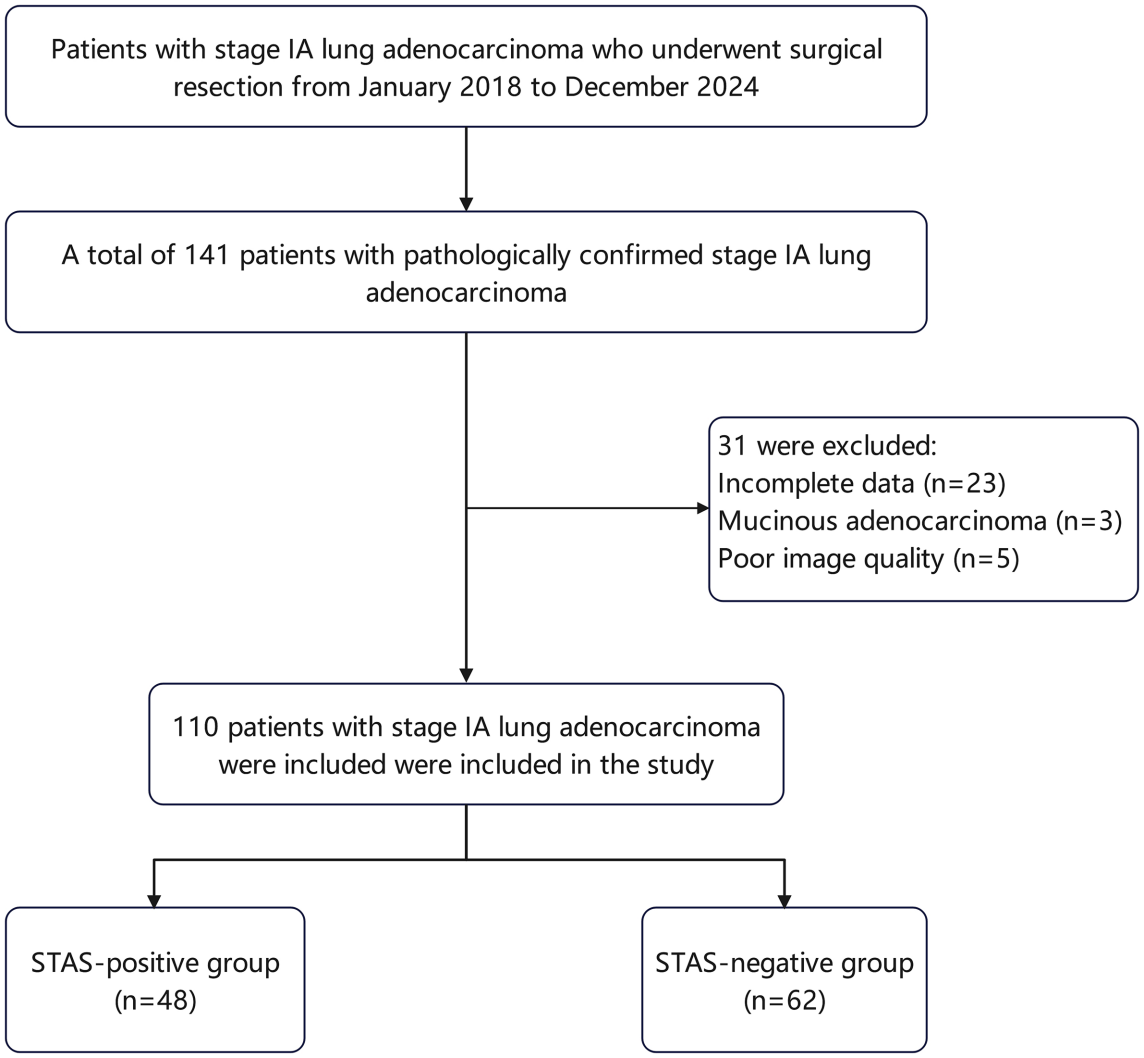
Flowchart of patient selection procedure. STAS, spread through air spaces.

This study was performed in line with the principles of the Declaration of Helsinki. The study was approved by our institution Research Ethics Committee (2023-NZR-100), and it was determined that informed consent was not required. The requirement for informed consent from participants was waived in accordance with the regulations. We retrospectively analyzed the clinical data of 141 patients with stage IA lung adenocarcinoma who underwent surgical treatment and were pathologically confirmed at our institution from May 2018 to December 2024 (Figure 1). The inclusion criteria were as follows: (1) patients diagnosed with stage IA lung adenocarcinoma after surgery at our hospital; (2) pathological confirmation of STAS status; (3) the interval between the preoperative CT scan and surgery did not exceed 2 weeks; (4) patients had not received any form of radiotherapy or chemotherapy prior to surgery. The exclusion criteria included: (1) poor quality of preoperative chest CT images due to various artifacts; (2) pathological diagnosis of mucinous adenocarcinoma; (3) missing imaging, pathological, or clinical data. A total of 110 patients participated in this study. The STAS-positive group consisted of 48 patients, including 15 males and 33 females, with a mean age of 60.60 ± 9.18 years. The STAS-negative group included 62 patients, comprising 19 males and 43 females, with a mean age of 60.60 ± 7.52 years.

### CT scanning and image acquisition

**Figure 2:**
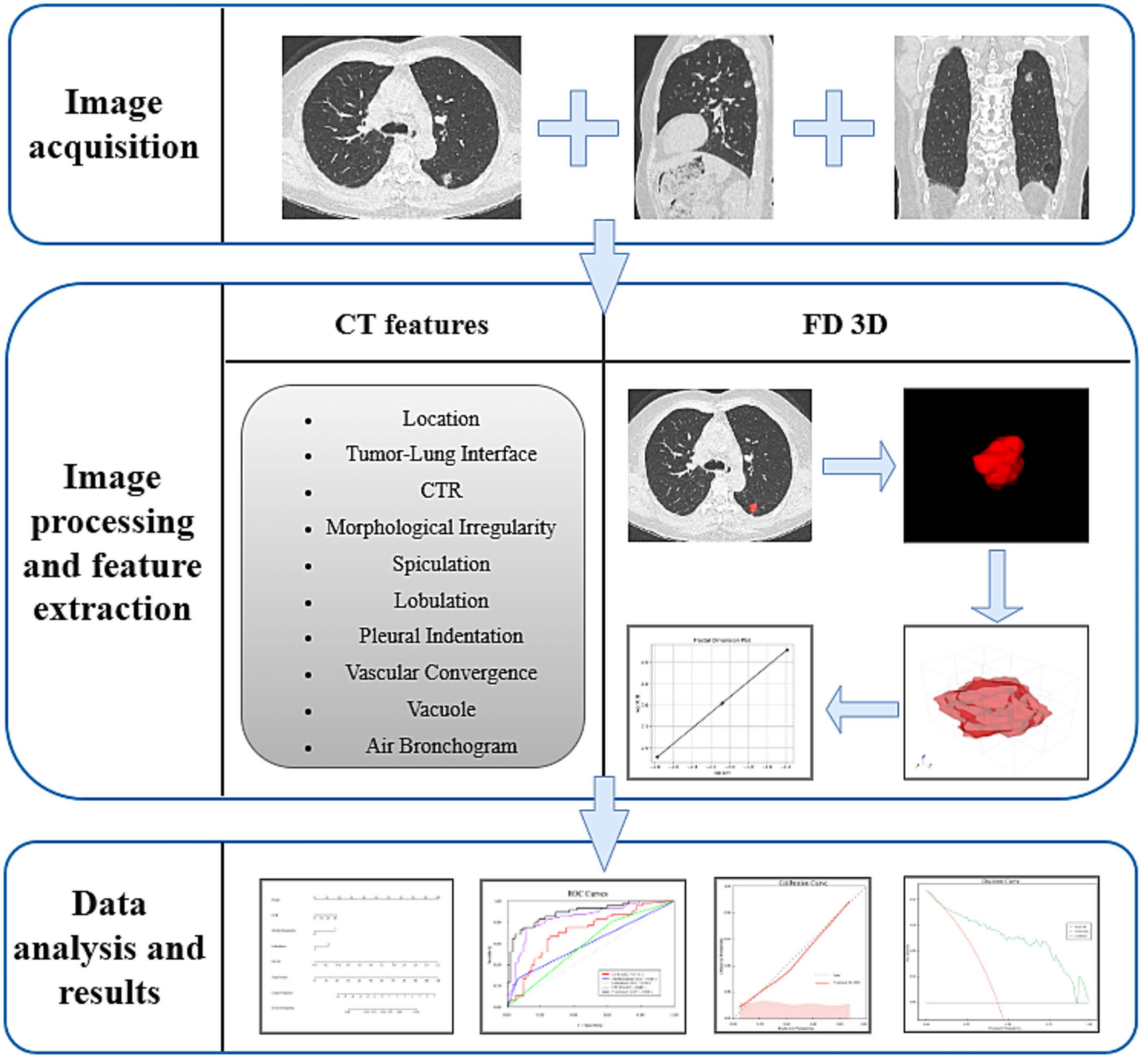
Overview of the research pipeline. CTR, consolidation tumor ratio; FD 3D, three-dimensional fractal dimension.

All patients underwent chest CT scans within 2 weeks prior to surgery. Scanning was conducted using a single 256-row wide-detector CT scanner (Revolution, GE Healthcare, USA). The scans were performed in the supine position with deep inspiration and arms elevated, covering the area from the thoracic inlet to the posterior costophrenic angle. The scanning parameters were configured as follows: tube voltage at 120 kV, tube current ranging from 100 to 150 mA, beam pitch of 1.375 or 1.5, and slice thickness at 0.625 mm, with a noise index (NI) of 20. The standard reconstruction algorithm applied was the adaptive statistical iterative reconstruction (ASIR-V) algorithm with a reconstruction kernel of standard (ST) for image reconstruction. Two physicians with 8 and 10 years of clinical experience in thoracic imaging independently reviewed and analyzed the imaging features. The consolidation tumor ratio (CTR) was extracted from the PACS system and sent to the lung nodule artificial intelligence analysis system for automatic identification and extraction of lung nodules. During the evaluation process, if disagreements arose between the two physicians, they would reach a consensus through in-depth discussion. Furthermore, both physicians were blinded to the presence of STAS during the assessment (see Figure 2).

### Fractal dimension delineation and extraction

#### Fractal dimension analysis

Fractal dimension analysis was conducted to quantitatively characterize the morphological complexity of tumors. The FD 2D was calculated using ImageJ software (9) (National Institutes of Health, Bethesda, MD, USA). For FD 3D, we developed a fully custom implementation in Python (version 3.10) based on the classical three-dimensional box-counting algorithm.

#### ROI segmentation

Tumor segmentation was performed manually using 3D Slicer software (version 5.8.1). For each case, the tumor boundaries were delineated slice-by-slice on axial CT images using lung window settings (window level: −600 HU; window width: 1500 HU). The entire three-dimensional tumor volume was incorporated into the region of interest (ROI) to ensure comprehensive morphological characterization. All segmentations were performed independently by two board-certified radiologists with specialized expertise in thoracic imaging.

### Image preprocessing

Following segmentation, ROI masks were exported in NIfTI format for subsequent analysis. Voxel spacing information from the original CT images was preserved intact. No spatial resampling, smoothing, or interpolation was applied to prevent any alteration of the intrinsic tumor geometry. Prior to fractal analysis, tumor masks were binarized, with tumor voxels assigned a value of 1 and background voxels assigned a value of 0.

### 3D box-counting algorithm

FD 3D was computed using our custom Python implementation of the classical three-dimensional box-counting algorithm. The computational workflow comprised the following steps:

1. The binarized three-dimensional tumor volume was embedded within a regular spatial grid;
2. A series of cubic boxes with progressively increasing sizes were deployed to cover the tumor volume;
3. At each scale, the number of boxes containing at least one tumor voxel was enumerated;
4. Box counts corresponding to different box sizes were systematically recorded;
5. A log–log plot was constructed relating the inverse box size to the corresponding box count;
6. Linear regression was applied to the log–log data, with the slope of the fitted regression line defined as the FD 3D value.

Identical box size ranges and scaling strategies were employed across all cases to ensure measurement comparability. Higher FD 3D values reflect increased spatial complexity and geometric irregularity of the tumor volume.

### Visualization and quality control

**Figure 3:**
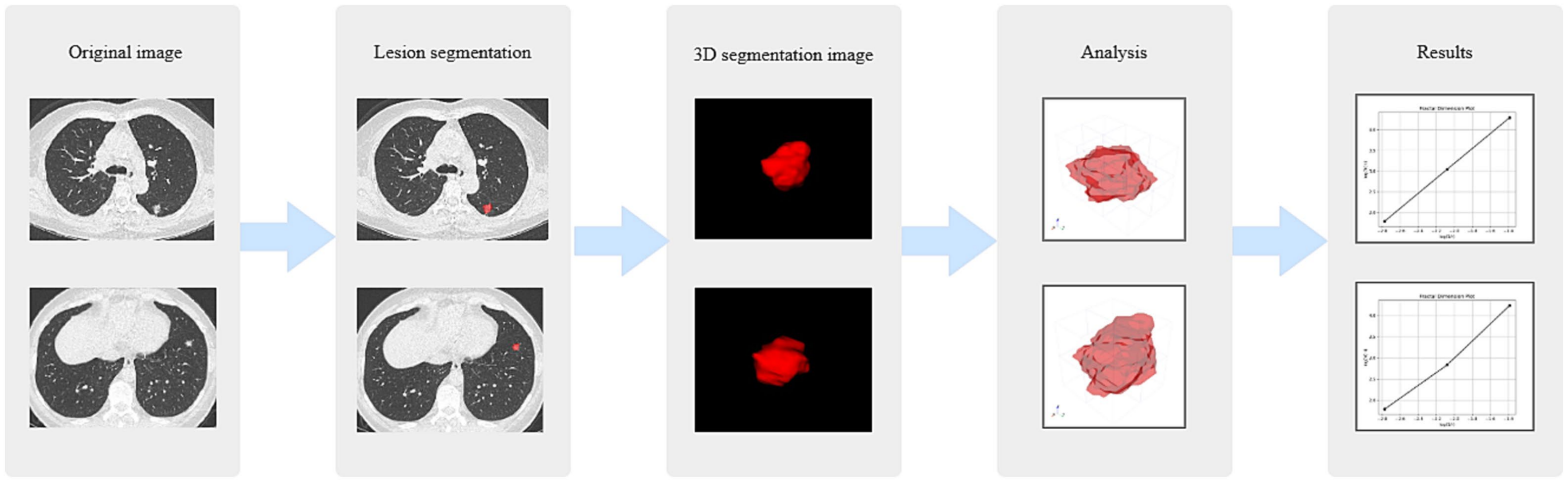
FD 3D analysis workflow. FD 3D, three-dimensional fractal dimension.

To facilitate qualitative assessment of the box-counting process, representative three-dimensional box coverage maps were generated for visualization purposes. Additionally, the log–log fitting curves utilized for FD estimation were archived to verify the linearity of scaling behavior (Figure 3).

### Reproducibility assessment

To evaluate the reproducibility of FD 3D measurements, 20 cases were randomly selected and re-segmented by the same two radiologists after a four-week interval. Inter-observer and intra-observer agreement were quantified using the intraclass correlation coefficient (ICC). The resulting ICC values were 0.92 for ROI volume and 0.91 for FD 3D, demonstrating excellent reproducibility for both segmentation and quantitative measurements.

### Pathology

**Figure 4:**
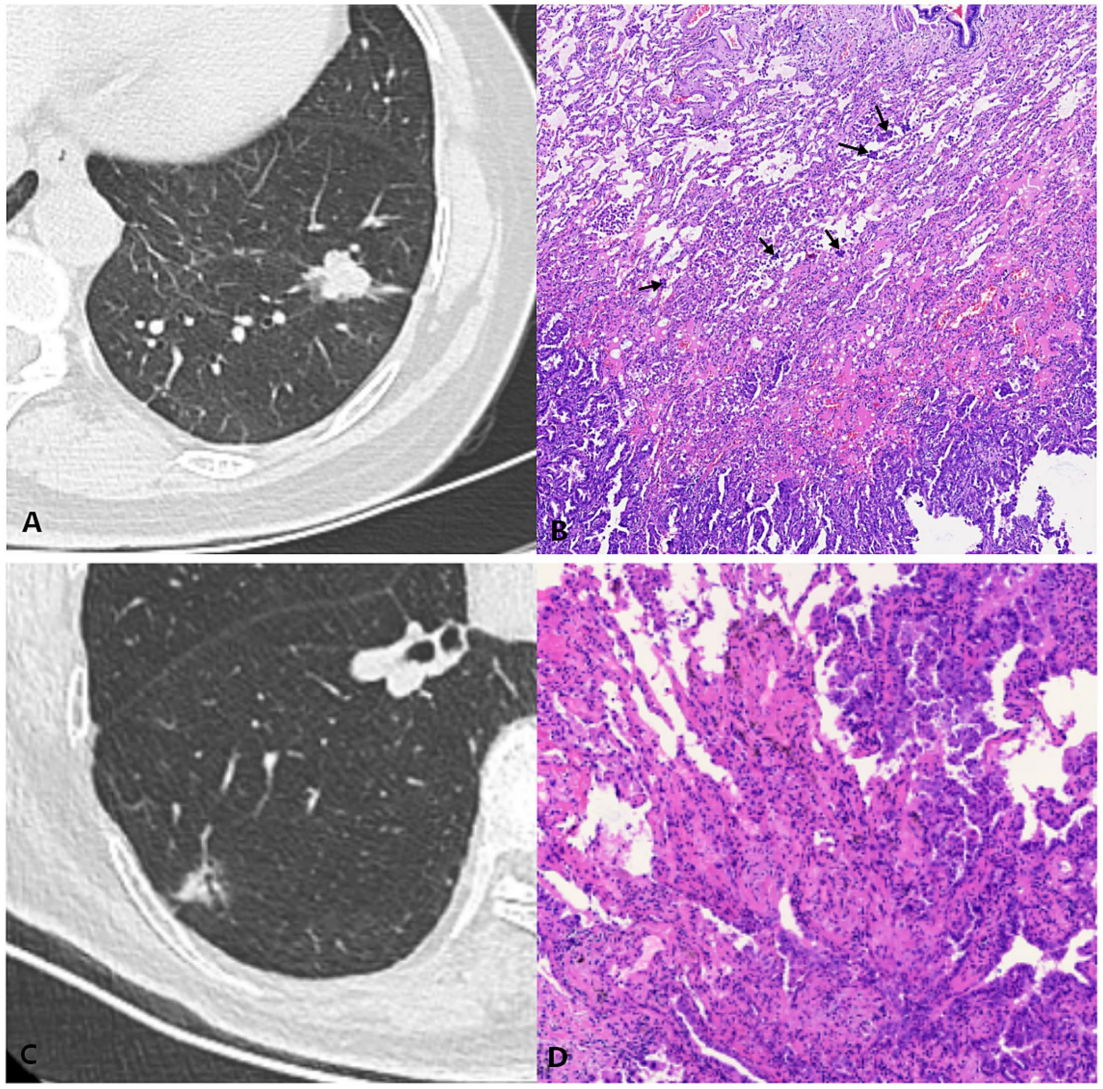
Computed tomography (CT) and histology images of lung adenocarcinoma with and without spread through air spaces (STAS). **(A,B)** STAS-positive case: **(A)** Axial CT image (window width, 1,500 HU; window level, −600 HU) shows a solid nodule; **(B)** Photomicrograph (hematoxylin–eosin stain) shows detached micropapillary clusters and solid nests of tumor cells in alveolar spaces beyond the edge of the main tumor. **(C,D)** STAS-negative case: **(C)** Axial CT image (window width, 1,500 HU; window level, −600 HU) shows a mixed ground-glass opacity nodule; **(D)** Photomicrograph shows the absence of detached tumor cell clusters in alveolar spaces beyond the main tumor edge.

All data in this study were meticulously extracted and organized based on the surgical pathology reports. Two pathologists, each with extensive experience at or above the level of deputy chief physician, conducted a thorough review of the hematoxylin–eosin-stained tissue sections. This article strictly adhered to WHO’s explicit definition of tumor STAS, without knowledge of the patients’ clinical outcomes (4) (Figure 4).

### Statistical analysis

Statistical analysis was conducted using SPSS version (version 27), and R language (version 4.3.3), with a significance level of *p* < 0.05. For data that followed a normal distribution, the mean ± standard deviation ( $\bar{x}$ ±s) was used for description, and intergroup comparisons were made using independent sample t-tests. Conversely, for data that did not conform to a normal distribution, the median (interquartile range) was employed for description, and the Mann–Whitney U test was used for intergroup comparisons. Count data were described using frequencies and percentages, and intergroup comparisons were performed using the *χ*^2^ test or Fisher’s exact test. Univariate logistic regression analysis was conducted to compare differences between the two groups. The variance inflation factor (VIF) was employed to assess multicollinearity among the independent variables. For variables with *p* values less than 0.05 in the univariate analysis, multivariate logistic regression analysis was conducted to identify independent predictors. Receiver operating characteristic (ROC) curves were generated to evaluate both the independent predictors and the combined model. Model calibration was assessed using the Hosmer–Lemeshow test and bootstrap resampling (1,000 iterations). The “rms” package in R was utilized to construct a nomogram based on the identified independent predictors. The model’s validity was assessed by plotting calibration curves and conducting decision curve analysis (DCA).

## Results

### Clinical, imaging, and fractal dimension features of patients

Significant differences were observed between the two groups regarding CTR, morphological irregularity, lobulation, pleural indentation, vascular convergence, and FD 3D (all *p* < 0.05). However, no significant differences were found in lesion location, tumor-lung interface, spiculation, vacuoles, and air bronchograms (all *p* > 0.05). Specific clinical, imaging, and fractal dimension features are presented in Table 1.

**Table 1:** Baseline clinical, imaging, and fractal-dimension characteristics of the study population.

Parameter	STAS(+) (*n* = 48)	STAS(−) (*n* = 62)	*χ*^2^/t/Z	*p*-value
Sex			0.005	0.946
Female	33 (68.75)	43(69.35)		
Male	15 (31.25)	19(30.65)		
Age	60.60 ± 9.18	60.60 ± 7.52	0.005	0.996
Smoking			0.807	0.369
Never	37 (77.08)	52 (83.87)		
Former/current	11 (22.92)	10 (16.13)		
Location			1.119	0.290
Upper and middle part of the lobe	27 (56.25)	41 (66.13)		
Lower part of the lobe	21 (43.75)	21 (33.87)		
Tumor-Lung interface			0.488	0.485
Clear	44 (91.66)	53 (85.48)		
Blurry	4 (8.33)	9 (14.52)		
CTR	50.30 (19.43,80.62)	16.96 (2.57,40.28)	−3.783	<0.001
Morphological irregularity			7.306	0.007
Absent	35 (72.92)	58 (93.55)		
Present	13 (27.08)	4 (6.45)		
Spiculation			0.956	0.328
Absent	29 (60.42)	43 (69.35)		
Present	19 (39.58)	19 (30.65)		
Lobulation			4.415	0.036
Absent	9 (18.75)	23 (37.10)		
Present	39 (81.25)	39 (62.90)		
Pleural indentation			8.461	0.004
Absent	16 (33.33)	38 (61.29)		
Present	32 (66.67)	24 (38.71)		
Vascular convergence			6.808	0.009
Absent	15 (31.25)	34 (54.84)		
Present	33 (68.75)	28 (45.16)		
Vacuole			0.296	0.586
Absent	35 (72.92)	48 (77.42)		
Present	13 (27.08)	14 (22.58)		
Air Bronchogram			1.730	0.188
Absent	25 (52.08)	40 (64.52)		
Present	23 (47.92)	22 (35.48)		
FD 2D	1.36 ± 0.08	1.36 ± 0.10	0.021	0.983
FD 3D	3.25 (3.11,3.32)	3.52 (3.43,3.70)	−6.172	<0.001

STAS, spread through air spaces; CTR, consolidation tumor ratio; FD 2D, two-dimensional fractal dimension; FD 3D, three-dimensional fractal dimension.

### Univariate and multivariate logistic regression analysis for predicting STAS

Univariate logistic regression analysis revealed that CTR [OR = 1.024, 95% CI (1.011, 1.037), *p* < 0.001], morphological irregularity [OR = 5.386, 95% CI (1.628, 17.820), *p* = 0.006], lobulation [OR = 2.556, 95% CI (1.050, 6.219), *p* = 0.039], pleural indentation [OR = 3.167, 95% CI (1.440, 6.965), *p* = 0.004], vascular convergence [OR = 2.862, 95% CI (1.285, 6.374), *p* = 0.010], and FD 3D [OR = 0.002, 95% CI (0.000, 0.021), *p* < 0.001] were significantly associated with STAS in stage IA lung adenocarcinoma (Table 2). Further multivariate regression analysis indicated that CTR [OR = 1.022, 95% CI (1.001, 1.044), *p* = 0.041], morphological irregularity [OR = 15.388, 95% CI (2.695, 87.299), *p* = 0.002], lobulation [OR = 0.190, 95% CI (0.038, 0.954), *p* = 0.044], and FD 3D [OR = 0.001, 95% CI (0.000, 0.023), *p* < 0.001] were independent predictors of STAS positivity. The associations of pleural indentation and vascular convergence with STAS were no longer significant (Table 2). Collinearity analysis of the four factors showed VIF values of 1.28, 1.13, 1.51, and 1.36, respectively, all less than 5, indicating no multicollinearity among these factors.

**Table 2:** Univariate and multivariate analysis results between groups.

Parameter	Univariate analysis			Multivariate analysis		
	OR	95% CI	*p*-value	OR	95% CI	*p*-value
Gender	1.029	0.455, 2.324	0.946	—	—	—
Age	1.000	0.955, 1.047	0.996	—	—	—
Smoking	1.546	0.595, 4.015	0.371	—	—	—
Location	0.659	0.303, 1.431	0.291	—	—	—
Tumor-Lung interface	0.535	0.154, 1.857	0.325	—	—	—
CTR	1.024	1.011, 1.037	<0.001	1.022	1.001, 1.044	0.041
Morphological irregularity	5.386	1.628, 17.820	0.006	15.388	2.695, 87.299	0.002
Spiculation	1.483	0.672, 3.271	0.329	—	—	—
Lobulation	2.556	1.050, 6.219	0.039	0.190	0.038, 0.954	0.044
Pleural indentation	3.167	1.440, 6.965	0.004	2.618	0.776, 8.831	0.121
Vascular convergence	2.862	1.285, 6.374	0.010	0.964	0.284, 3.266	0.953
Vacuole	1.273	0.533, 3.045	0.587	—	—	—
Air bronchogram	1.673	0.775, 3.609	0.190	—	—	—
FD 2D	1.047	0.015, 72.237	0.983	—	—	—
FD 3D	0.002	0.000, 0.021	<0.001	0.001	0.000–0.023	<0.001

CTR, consolidation tumor ratio; FD 2D, two-dimensional fractal dimension; FD 3D, three-dimensional fractal dimension; OR, odds ratio.

### Construction and performance evaluation of the nomogram model

**Figure 5:**
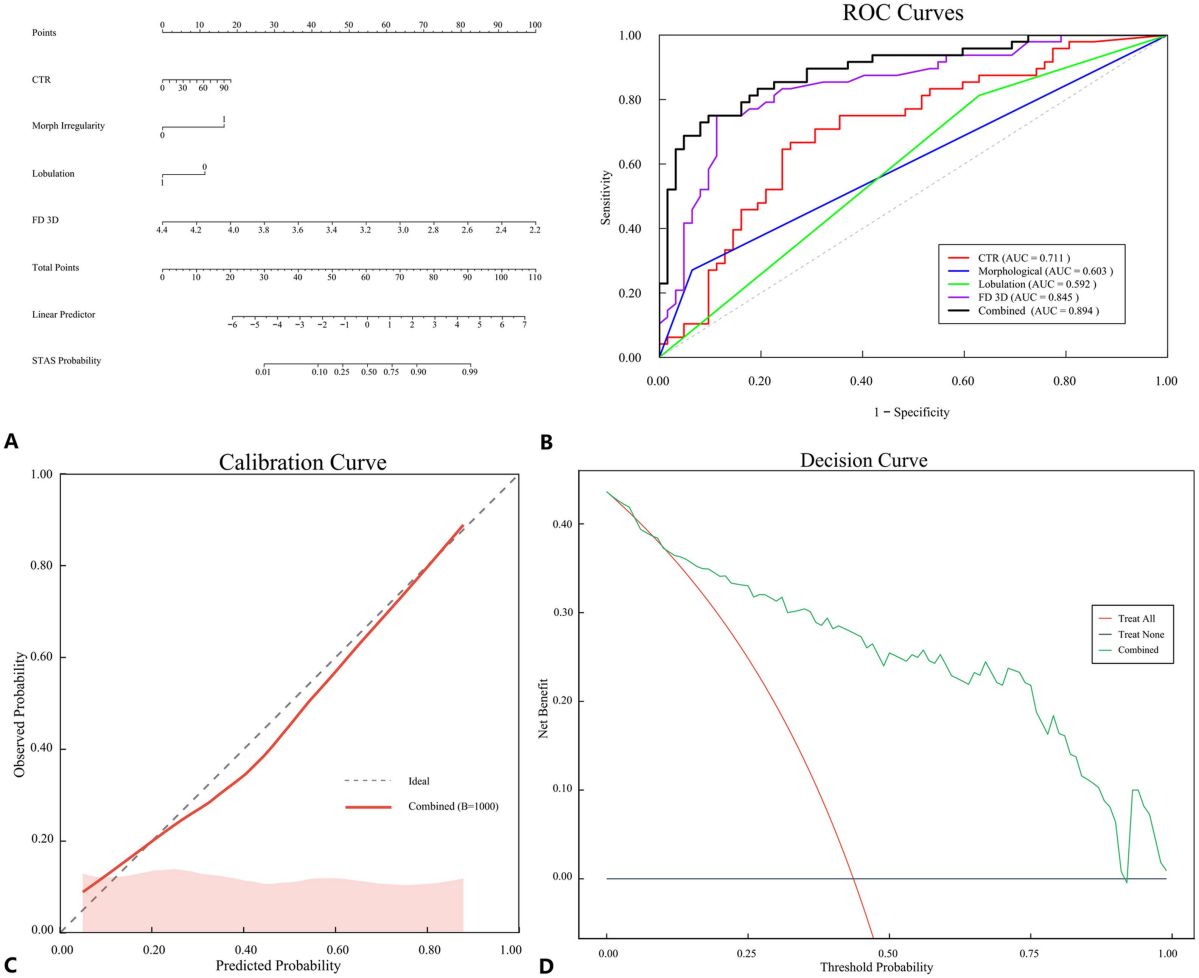
**(A)** A nomogram for the combined model; **(B–D)** Evaluation and verification of the combined model: **(B)** ROC curves of the combined model compared with CTR, morphological irregularity, lobulation, and FD 3D; **(C)** Calibration curve of the combined model was used to determine the predictive accuracy of STAS; **(D)** Decision curve analysis (DCA) showed the clinical usefulness of the combined model. STAS, spread through air spaces; CTR, consolidation tumor ratio; FD 3D, three-dimensional fractal dimension.

Based on the independent predictors identified through multivariate logistic regression analysis (CTR, morphological irregularity, lobulation, and FD 3D), a nomogram model for predicting STAS was constructed using R language. The regression equation was as follows: STAS numeric = −2.1 + 0.3 × CTR + 0.2 × morphological irregularity + 0.15 × lobulation + 0.4 × FD 3D. As shown in Figure 5A, each variable corresponds to a specific score, with the magnitude of the score reflecting the OR value of the variable, and the total score corresponding to the predicted probability of STAS positivity. The nomogram model demonstrated favorable predictive performance. The ROC curve analysis (Figure 5B) revealed an AUC of 0.894 (95% CI: 0.821–0.945; sensitivity 75.00%, specificity 90.32%), which was superior to that of CTR (AUC 0.711), morphological irregularity (AUC 0.603), lobulation (AUC 0.592), and FD 3D alone (AUC 0.844) (Table 3). Model calibration was assessed using the calibration curve, Hosmer–Lemeshow test, and bootstrap resampling (1,000 iterations). The calibration curve demonstrated good consistency between the predicted and actual risks of STAS positivity (Figure 5C). The Hosmer–Lemeshow test yielded a *p*-value of 0.322, and bootstrap validation confirmed the robustness of the model calibration, indicating good discrimination ability. Decision curve analysis (DCA) showed that the nomogram model provided good net benefit for patients within a threshold probability range of 10 to 80%, indicating its clinical applicability (Figure 5D). Furthermore, the nomogram could identify approximately 25% of patients who might benefit from lobectomy rather than sublobar resection; however, this observation should be regarded as hypothesis-generating and requires prospective validation.

**Table 3:** ROC curve analysis results.

Parameter	AUC	95% CI	Sensitivity (%)	Specificity (%)	*p*-value
CTR	0.711	0.617, 0.793	66.67	74.19	<0.001
Morphological irregularity	0.603	0.505, 0.695	27.08	93.55	0.004
Lobulation	0.592	0.494, 0.684	81.25	37.10	0.03
FD 3D	0.845	0.763, 0.907	75.00	88.71	<0.001
Combined	0.894	0.821, 0.945	75.00	90.32	<0.001

CTR, consolidation tumor ratio; FD 3D, three-dimensional fractal dimension; AUC, area under the curve.

### Risk stratification and clinical utility

To further evaluate the clinical utility of the nomogram, we performed risk stratification analysis based on the optimal cutoff value of 0.56 (determined by the maximum Youden index). Patients were divided into high-risk (*n* = 42, 38.18%) and low-risk (*n* = 68, 61.82%) groups. The high-risk group demonstrated a PPV of 85.71% (36/42) for STAS positivity, while the low-risk group showed an NPV of 82.35% (56/68) (Table 4). The prevalence of STAS differed significantly between the two groups (85.71% vs. 17.65%, *χ*^2^ = 46.18, *p* < 0.001), indicating effective risk stratification. These results suggest that the nomogram could identify approximately 38% of patients who would benefit from lobectomy (high PPV) and 62% of patients who might be candidates for sublobar resection (acceptable NPV).

**Table 4:** Predictive performance of the nomogram in risk-stratified groups.

Group	*N*	STAS positive	STAS prevalence (%)	PPV (%)	NPV (%)
Overall	110	48	43.64	—	—
High-risk (≥0.56)	42	36	85.71	85.71	—
Low-risk (<0.56)	68	12	17.65	—	82.35

STAS, spread through air spaces; PPV, positive predictive value; NPV, negative predictive value.

## Discussion

Large-scale studies have consistently shown that STAS-positive lung adenocarcinoma is associated with higher recurrence risk after limited resection, and that a wider resection extent may improve outcomes compared with STAS-negative disease. Accordingly, accurate preoperative identification of STAS has become clinically important, especially in stage IA patients where the choice between sublobar resection and lobectomy is often debated (5, 10–14). However, most existing CT-based approaches rely heavily on intensity- or texture-derived parameters, which are sometimes difficult to interpret biologically and may not fully capture the growth patterns underlying STAS. There remains a need for imaging biomarkers that are both predictive and mechanistically plausible.

In this context, we built a preoperative CT nomogram for predicting STAS in stage IA lung adenocarcinoma by integrating conventional imaging features with FD 3D. Rather than replacing established CT markers, FD 3D was intended to provide an additional geometric descriptor of tumor morphology that is less dependent on gray-level statistics and potentially easier to relate to tumor growth patterns.

Fractal dimension offers a quantitative way to describe the geometric complexity of biological structures. In lung cancer imaging, prior work has suggested that FD-related metrics may help differentiate invasive from less invasive lesions, and FD-based models have shown value in characterizing invasiveness in ground-glass nodules (15). Nevertheless, the role of FD—particularly in a fully three-dimensional form—for preoperative assessment of STAS has been less explored.

We did not observe a significant association between FD 2D and STAS status. One plausible explanation is that two-dimensional measurements are inherently sensitive to slice selection and may fail to reflect the three-dimensional spatial characteristics of early-stage tumors. In addition, STAS can present with a discontinuous or “skip” pattern of spread, which may not be adequately represented on a single axial section (16). These factors may reduce the sensitivity of planar FD estimates and partly explain the negative 2D findings in our cohort.

FD 3D showed good discriminative performance in our cohort (AUC = 0.844; sensitivity = 75.0%; specificity = 88.7%) and was significantly lower in STAS-positive tumors. This direction is biologically plausible if FD 3D is viewed as a measure of three-dimensional spatial continuity rather than simply “roughness.” Pathologically, STAS-positive tumors often exhibit discontinuous growth with scattered tumor cell clusters and areas of necrosis, producing a fragmented architecture (17). Such fragmentation can reduce the global spatial continuity of the tumor mass on imaging, which may be captured as a lower FD 3D. Fan et al. (18) further linked the scattered distribution of STAS tumor cells to hypoxia-related migration; this type of heterogeneous, discontinuous growth could likewise contribute to a reduced fractal dimension in three dimensions. Importantly, even in small stage IA tumors, the presence of STAS reflects invasive potential, and FD 3D may serve as a quantitative surrogate of this “discontinuous” growth phenotype.

CTR, which quantifies the proportion of the solid component within a lung adenocarcinoma, has been associated with multiple invasive behaviors including lymph node metastasis, vascular invasion, and pleural invasion (19). Several studies have also reported a relationship between higher CTR and STAS positivity (20–24). For example, Qi et al. (22) showed that CTR was independently associated with STAS (AUC = 0.760), and Liu et al. (24) found CTR to be an important feature in a random forest model for stage IA disease. In our cohort, CTR demonstrated moderate performance (AUC = 0.711). Its specificity (74.19%) suggests that CTR alone is susceptible to confounding factors and is better interpreted as one component of a multivariable model rather than a stand-alone marker.

Morphological irregularity was independently associated with STAS and showed high specificity (93.55%) but limited sensitivity (27.08%) in our dataset. Clinically, this pattern suggests that marked irregularity, once present, is more useful for “ruling in” STAS than for screening. When incorporated into the nomogram, this high-specificity feature contributed to the overall model specificity (90.32%). Lobulation also emerged as an independent predictor, with relatively higher sensitivity (81.25%) but lower specificity (37.10%), indicating a complementary role in screening. Taken together, combining a sensitive marker (lobulation) with a specific marker (morphological irregularity) may improve practical risk stratification, which is consistent with the concept that STAS represents a more aggressive invasion phenotype and often coexists with irregular tumor margins (25, 26).

Methodologically, our work represents an application of three-dimensional fractal analysis to STAS prediction. Compared with conventional radiomic features that primarily summarize intensity distributions and textures (27), FD 3D provides a geometry-oriented measure that can be interpreted in relation to tumor continuity and fragmentation. This offers a more direct bridge between macroscopic morphology on CT and the discontinuous growth behavior observed pathologically in STAS. Future studies may explore whether combining FD 3D with molecular information can further improve risk stratification, but such integration should be evaluated in well-designed multicenter cohorts.

The nomogram demonstrated favorable PPV (85.71%) and NPV (82.35%) in risk-stratified analysis. The high PPV in the high-risk group suggests potential utility for identifying patients who might benefit from definitive lobectomy, given that more than 6 out of 7 high-risk patients truly had STAS. Meanwhile, the acceptable NPV (82.35%) in the low-risk group indicates potential for selecting sublobar resection candidates, with approximately 4 out of 5 low-risk patients being truly STAS-negative. However, we acknowledge that an NPV of ~82% requires cautious interpretation, as missing nearly 1 in 5 STAS cases could have significant oncologic consequences. Therefore, while these findings suggest potential clinical utility, the application of FD 3D to guide surgical extent remains a hypothesis requiring prospective validation before routine clinical implementation.

This study has several limitations. First, its retrospective single-center design may introduce selection bias. Second, tumor regions of interest were manually delineated using 3D Slicer, and inter-observer variability may affect measurement stability despite the use of a standardized segmentation workflow. Third, the predictive model was internally validated, and external validation is still required before broader clinical application.

Future work will focus on prospective multicenter validation to assess the robustness and generalizability of the model across different institutions and CT vendors. In addition, the development of automated or semi-automated segmentation tools may further improve reproducibility and facilitate clinical implementation. Finally, integrating FD 3D with molecular biomarkers, such as EGFR or ALK status, may provide additional insight into the biological basis of STAS and warrants further investigation.

## Conclusion

In conclusion, CTR, morphological irregularity, lobulation, and FD 3D were identified as independent predictors of STAS in stage IA lung adenocarcinoma. By integrating these parameters, we developed a CT-based nomogram that enables noninvasive preoperative risk assessment of STAS. This geometry-oriented approach complements conventional imaging features and may assist thoracic surgeons in tailoring the extent of resection for individual patients.

## Data Availability

The raw data supporting the conclusions of this article will be made available by the authors, without undue reservation.
